# Evaluation of the Effect of Early-Onset Steroid Treatment in the COVID-19-Positive Pregnant Women on Pregnancy Outcomes

**DOI:** 10.3390/v16091453

**Published:** 2024-09-12

**Authors:** Neval Elgormus, Abdulhalim Senyigit, Omer Okuyan, Fatma Bozkurt, Derya Sivri Aydin, Hafize Uzun

**Affiliations:** 1 Department of Microbiology, Faculty of Medicine, Istanbul Atlas University, Istanbul 34008, Turkey; neyelgormus@yahoo.com; 2 Department of Internal Medicine, Faculty of Medicine, Istanbul Atlas University, Istanbul 34008, Turkey; abdulhalim.senyigit@atlas.edu.tr; 3 Department of Child Health and Diseases, Faculty of Medicine, Istanbul Atlas University, Istanbul 34008, Turkey; dmemhs@gmail.com; 4 Department of Infectious Diseases and Clinical Microbiology, Faculty of Medicine, Istanbul Atlas University, Istanbul 34008, Turkey; drfatmayakut@hotmail.com; 5 Department of Obstetrics and Gynecology, Faculty of Medicine, Istanbul Atlas University, Istanbul 34008, Turkey; deryasivri@hotmail.com; 6 Department of Medical Biochemistry, Faculty of Medicine, Istanbul Atlas University, Istanbul 34008, Turkey

**Keywords:** SARS-CoV-2, COVID-19, pregnancy, preterm labor, corticosteroids, dyspnea

## Abstract

Objective: Coronavirus disease 2019 (COVID-19) is the disease caused by severe acute respiratory syndrome coronavirus 2 (SARS-CoV-2). Acute respiratory distress and preterm delivery are the two major complications induced by SARS-CoV-2 infection during pregnancy. In the presence of dyspnea, the use of systemic corticosteroids was recommended in pregnant and non-pregnant groups. Our primary aim was to investigate the effect of early-onset steroid treatment on mortality and adverse effects in pregnant women with COVID-19. Our secondary aim was to investigate the effect of steroid treatment on the length of hospital stay and intensive care unit (ICU) stay, and duration of treatment. The study also investigated infection, preterm birth, and ideal body weight (lbw) in newborns. Methods: In this retrospective study, 253 patients were divided into three groups according to steroid administration. In Group 1 patients (n:112), treatment was started at the time of hospitalization. In Group 2 patients (n:90), treatment was started at least 24 h after hospitalization. Group 3 consisted of patients (n:51) who did not receive steroid treatment. Methylprednisolone (32 mg/day) was given to pregnant patients with a gestational age below 24 weeks or above 34 weeks, and dexametazone (6 mg/day) was given in four doses followed by 32 mg/day methylprednisolone for the others (whose baby was at a gestational age of 24 weeks and above but less than 34 weeks). Result: The hospital stay, ICU stay, and steroid administration time were significantly lower in the Group 1 when compared to the others (*p* < 0.05). The steroid treatment requirement was 4.4 days in Group 1 and 5.7 days in Group 2 (*p* < 0.05). While no death was observed in Group 1, one patient died in Group 2 and three patients died in Group 3. There was no difference between the groups in terms of complications, including preterm labor. Conclusions: No death was also observed with early-onset treatment. Early-onset treatment may be beneficial for fewer hospitalizations, fewer ICU stays, and less mechanical ventilation requirement in pregnant women with COVID-19. In addition, with early treatment, the total number of steroid administration days was reduced, which is important in terms of reducing the risk of side effects.

## 1. Introduction

Severe acute respiratory syndrome coronavirus 2 (SARS-CoV-2), the cause of coronavirus disease 2019 (COVID-19), has become a very important public health problem due to its contagiousness and high death rate. Although this disease affects almost every age group, it is more severe especially in individuals with comorbidities [1]. Physiological changes in pregnancy make the individual more susceptible to lung damage [2,3,4]. Therefore, early and appropriate treatment gains importance. Besides this, preterm labor is another serious problem in COVID-19-positive pregnant woman [1].

The use of steroids was recommended for the patients with COVID-19 disease who require oxygen support [5]. Although the RECOVERY study did not include many pregnant women, it was recommended to use oral prednisolone or intravenous hydrocortisone for moderate and severe disease [5]. The use of steroids is recommended in pregnant women with moderate–severe disease; however, possible maternal, fetal, and postnatal side effects lead the clinician to a dilemma. Another important point is that the number of studies on this subject is very limited. In the past, steroid treatment was not applied to pregnant women who had moderate disease [6], but it was decided to start steroid treatment for patients who meet the criteria determined as a result of comprehensive studies [7,8,9]. In addition, it was thought that an early and short steroid treatment may be useful for fetal lung development [10,11].

In the absence of effective antiviral therapy, the early initiation of steroids is important in pregnant women as well as in non-pregnant adults. The RECOVERY study [5] included a low number of pregnant COVID-19 patients (n:6). It recommended that low-dose dexamethasone be used to reduce mortality rates in patients on mechanical ventilation. On the other hand, oral prednisolone or intravenous hydrocortisone was recommend for pregnant women. Another guideline recommended this therapy to pregnant women with moderate–severe disease [7]. Saad et al. [9] suggested oxygen support with methylprednisolone and/or dexamethasone according to the fetal lung maturation. 

Since SARS-CoV2 is an infection with a virus, it will be important to closely monitor the outcomes of both mothers and children born to mothers infected with it before and during pregnancy. It is also suggested that the health outcomes of children whose fathers were infected before conception should also be monitored [12]. Due to physical limitations and emotional distress during the COVID-19 lockdown, pregnant women may have a higher body mass index (BMI) and an increased diagnosis of gestational diabetes mellitus (GDM) [13]. Although vaccination and social isolation worldwide will contribute to curbing viral spread, millions of newborn babies have already been exposed to a virus with unknown consequences. In this sense, further studies should evaluate the actual effects of SARS-CoV2 infection during pregnancy and birth, as well as include a long-term follow-up of children, including neurocognitive, neuroimaging and electrophysiological examination [14]. 

Our primary aim was to investigate the effect of early-onset steroid treatment on mortality and adverse effects in pregnant women with COVID-19. Our secondary aim was to investigate the effect of steroid treatment on the length of hospital stay, intensive care unit (ICU) stay, and duration of treatment. The study also investigated infection, preterm birth, and ideal body weight (lbw) in newborns. 

## 2. Materials and Methods

This retrospective study was conducted at Department of Infectious Diseases and Clinical Microbiology, Medicine Hospital, Istanbul Atlas University, and Department of Infectious Diseases and Clinical Microbiology, Faculty of Medicine, University of Health Science, Gazi Yaşargil Education and Research Hospital, Diyarbakır, Turkey. The protocol for sample collection was approved by the Medicine Hospital at Istanbul Atlas University (Number: E-22686390-050.99-26198; Date: 12 April 2023). The study was performed in accordance with the Helsinki Declaration. Since the study was retrospective, the informed consent form was waived. 

### 2.1. Study Design

This retrospective study included pregnant women who were diagnosed with COVID-19 in a tertiary pandemic center between 1 March 2020 and 30 April 2022. Individuals who came to our clinic with dyspnea and had positive COVID-19 PCR test were included in the study. The lungs were evaluated and graded according to progression using computed tomography (CT). All pregnant women were unvaccinated.

### 2.2. Inclusion Criteria

By direction of COVID-19 diagnosis guideline, women with severe and/or critical diagnosed COVID-19 who applied to the Emergency Department or Infectious Diseases who were older than 18 years of age, who had their lungs evaluated by CT imaging and who had sufficient data to diagnose COVID-19 were included in this study. Patients hospitalized in the infectious diseases clinic were included in the study. Patients with positive COVID-19 PCR test, respiratory distress, low oxygen saturation despite 2 h of oxygen therapy, and involvement on chest CT were also included in the study. 

### 2.3. Exclusion Criteria

The following list of factors was excluded:

- Pregnant women with comorbid diseases such as hypertension, asthma, gestational diabetes, gestational cholestasis, pre-eclampsia, and atypical hemolysis, elevated liver enzymes, and low Platelets (HELLP) syndrome, and taking corticosteroids for any reason;
- Patients who were started on steroids before being diagnosed with COVID-19 due to the risk of preterm delivery;
- Patients who refused CT were excluded from the study. Pregnant women who conceived with in vitro fertilization (IVF), those with multiple pregnancies, and women who completed the data collection form incompletely and/or incorrectly were also excluded from the study;
- Patients with steroid administration after admission to the ICU due to critical disease were not considered as steroid-administered patients in this study.

Patients’ time interval between symptom onset and admission to the hospital, vital signs, oxygen saturation, gestational age, BMI, COVID-19-related symptoms with chest CT findings, treatment protocol, clinical follow-up information, and steroid-related side effects were retrospectively obtained from patients’ medical records. 

### 2.4. Use of Corticosteroids

At the beginning of the COVID-19 pandemic, there were no recommendations regarding the treatment of COVID-19-positive pregnant patients. In addition, WHO did not recommend steroid treatment in moderate-group COVID-19 patients. However, we started to use steroids in our patients after the data on good response to steroid treatment in moderate-group COVID-19 pregnant women (n:6) in the RECOVERY study were published [5]. Steroid treatment was started for the patients who met the following criteria at the time of admission of the patient: presence of dyspnea, CT findings compatible with COVID-19, respiratory rate of 30 or more per minute, peripheral oxygen saturation below 94, and under oxygen support with a venturi mask over 5 L/min. Methylprednisolone (32 mg/day) was given to pregnant patients with a gestational age below 24 weeks or above 34 weeks, and dexametazone (6 mg/day) in four doses followed by 32 mg/day methylprednisolone for the other pregnant patients (with gestational age of 24 weeks and above or those with gestational age of less than 34 weeks). It was decided to discontinue the steroid treatment with symptom relief. If the saturations were above 95% in room air without oxygen support for 24 h and there was no obstetric complication after one-day follow-up, discharge was planned. Obstetric complications were defined as complaints of abdominal contractions, groin pain, vaginal bleeding, and not feeling the baby’s movements.

Hyperglycemia developed as a side effect in patients receiving steroids. Blood glucose dysregulation that may occur following steroid administration was followed and insulin was administered to the patients when necessary. Symptomatic supportive treatment was given with paracetamol for patients with fever, I.V. fluids in dehydrated patients, and antiemetics in patients with vomiting. No antibiotics and/or antivirals other than steroids were given. Daily non-stress tests (NSTs) were performed for fetal follow-up. In the presence of decreased fetal movements, fetal biophysical profile and fetal cardiac examinations were performed. The chest CTs of the patients were evaluated by two experienced radiologists and they were classified into four grades: early, progression, peak, and resolution [15]. According to clinical findings and chest CT results, World Health Organization (WHO) classified the disease as mild, moderate, severe, and critical stage [6]. 

Two hundred fifty-three patients were divided into three groups according to steroid administration. In our hospital, the decision to start steroids in COVID-19 patients was administered in consultation with an infectious diseases specialist, a perinatology specialist, and a neonatologist [16]. Group 1 patients’ treatment was started at the time of hospitalization (within first 24 h). Group 2 patients’ treatment could be started at least 24 h after hospitalization, as they were waiting for the necessary investigations to be completed (24 h and later). Group 3 consisted of patients who did not receive treatment. During follow-up, those who needed intensive care due to acute respiratory failure were hospitalized in the ICU. The decision whether or not to admit the patients to the ICU was made by the anesthesiology and reanimation specialist in charge on that day after evaluation of the clinical and laboratory results of the patient. 

Pregnancy outcomes were evaluated as mean length of hospital stay, percentage of ICU stay, and number of days of ICU stay, neonatal infection, vertical transmission from the mother to the fetus or neonate, premature delivery, and ideal body weight (lbw). Preterm birth was defined as birth between 20^0/7^ weeks of gestation and 36^6/7^ weeks of gestation [17]. In addition, two consecutive COVID-19 PCR test results obtained from newborns at the time of delivery and within 24–48 h, and the fifth-minute APGAR score and birth weight records were examined. Clinical follow-up and observation data of newborn babies were evaluated.

### 2.5. Statistical Analysis

Statistical analysis was performed using SPSS version 22 (IBM, Armonk, NY, USA). The sample size of the study was calculated using G*Power 3.1.9.6 program. Categorical data were expressed as numbers and percentages. Numerical data were expressed as mean ± standard deviation. The Kolmogorov–Smirnov test was used to decide whether the samples came from a population with a particular distribution. Categorical variables were evaluated with chi-square test. The differences between the groups were analyzed with one-way Anova test. Games–Howell and Scheffe tests was used for post hoc analysis. The values less than 0.05 were considered to be statistically significant.

## 3. Results

**Figure 1:**
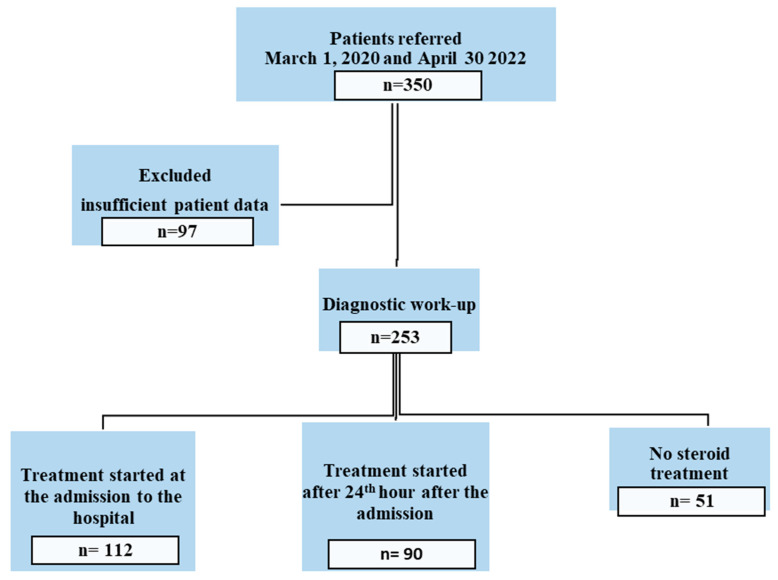
A flow chart of the selection of cases.

In total, 253 of the 350 COVID-19-positive patients were included in the study because they (253 patients) met the inclusion criteria (Figure 1). There were 112 (44.3%) patients in Group 1, 90 (35.6%) patients in Group 2, and 51 (20.2%) patients in Group 3. The mean age in the groups were 29.1, 31.1, and 29.7, respectively (*p* = 0.139). The mean day between symptom onset and admission to the hospital, mean BMI, and mean gestational age were similar between the groups. The percentage of severe COVID-19 illness was 93% in all patients (*p* = 0.609) (Table 1). The steroid treatment requirement was 4.4 days in Group 1 and 5.7 days in Group 2 (*p* < 0.001). The frequency of the increased blood glucose levels related to the steroid administration was similar in the groups (*p* = 0.657) (Table 2).

The mean hospital stay was 6 days in Group 1, 9 days in Group 2, and 11 days in Group 3 patients (*p* < 0.05). The rates of admission to the ICU due to respiratory deterioration were 7 (6.3%) in Group 1, 19 (21.1%) in Group 2, and 15 (29.4%) in Group 3 (*p* < 0.05). Besides this, the requirement of mechanical ventilation support was lowest in the Group 1, while the highest rate was in the Group 3 (*p* < 0.05). The stay day in the ICU in the groups was 2.7, 5.7, and 8.2, respectively, and the difference between the groups was statistically different (*p* < 0.05) (Table 2). We observed the death of four of the patients who were followed up with an invasive mechanical ventilator due to multiorgan failure, and these patients were included in Group 2 (n:1) and Group 3 (n:3). No death was observed in patients who received early-onset steroid therapy.

In the COVID-19 PCR screening of 89 newborns, the first COVID-19 RT-PCR test taken as soon as one baby was born was positive in Group 3 (the APGAR score was 9, and the girl was 3450 g). There was no statistically significant difference between the groups in the rate of preterm labor, low birth weight, average birth weight, and decrease in baby movements (*p* = 0.992) (Table 2). 

## 4. Discussion

**Figure 2:**
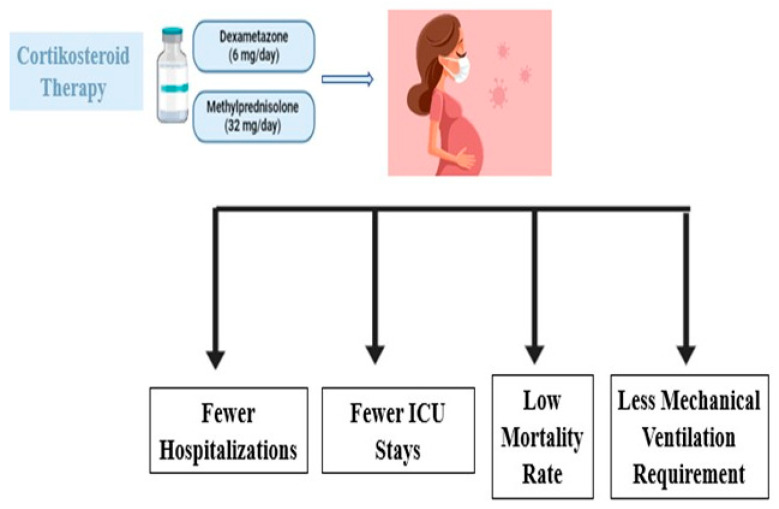
Pregnancy, coronavirus disease 2019 (COVID-19), and the effect of early-onset steroid treatment on pregnancy outcomes.

In current study, the hospital stay, ICU stay, and steroid administration time were significantly lower in Group 1 when compared to the others. The steroid treatment requirement was 4.4 days in Group 1 and 5.7 days in Group 2. While no death was observed in Group 1, one patient died in Group 2 and three patients died in Group 3. There was no difference between the groups in terms of complications, including preterm labor. To the best of our knowledge, this is the first report to show that early-onset steroid treatment may be beneficial for fewer hospitalizations, fewer ICU stays, and less mechanical ventilation requirement (Figure 2). Corticosteroid treatment in pregnancy should be carefully evaluated in terms of possible risks and the pregnant patient should be informed about this issue. We believe that corticosteroid therapy may be considered as a therapy especially in unvaccinated pregnant women with severe COVID-19. 

COVID-19 infection in pregnant women has been associated with higher caesarean section and mortality rates [18,19]. At the beginning of the outbreak, high mortality was predicted in pregnant women, but the mortality rate was not much higher than in non-pregnant women. As in non-pregnant people, mortality is high in the severe group in pregnant women. Furthermore, maternal mortality is mostly seen in those with pre-existing comorbidities, while neonatal mortality appears to be a consequence of prematurity rather than infection [20]. In the case of COVID-19 infection in pregnant women, most hospital admissions occurred during pregnancy, while death occurred in the postnatal period. Maternal obesity and maternal diabetes were risk factors in increasing the risk for COVID-19 disease progression in pregnant patients, resulting in an increased likelihood of ICU admission. Therefore, it is necessary to obtain more knowledge to pursue the earlier identification of pregnant women at a higher risk for severe COVID-19 disease [21].

We applied a similar treatment protocol to pregnant women who had moderate and severe COVID-19 disease. Studies support the use of methylprednisolone instead of prednisolone among pregnant women with severe COVID-19 [8]. In the current study, methylprednisolone (32 mg/day) was given to patients with a gestational age below 24 or above 34 weeks, and dexametazone (6 mg/day) in four doses followed by 32 mg/day methylprednisolone for the others (24 and above, to less than 34 weeks). Besides this, we observed that early-onset treatment in the first 24 h reduced the hospital stay, ICU stay, and day time of steroid administration without any fetal and postnasal complication. We did not observe any deaths due to COVID-19 when early-onset steroid therapy was administered. We observed the death of four of the patients who underwent invasive mechanical ventilation due to multiorgan failure, and these patients were included in Group 2 and Group 3. Studies have shown that only corticosteroids have been shown to definitively improve mortality in COVID-19 patients [22]. At the same time, the National Institutes of Health (NIH) have recommended, in their COVID-19 treatment guidelines, the use of dexamethasone in pregnant women with COVID-19 who are mechanically ventilated or who require supplemental oxygen, given the potential benefit of decreased maternal mortality and the low risk of fetal adverse effects associated with this short course of therapy [23]. Our results support these findings. These two drugs are not only cheaper in most low-resource/resource-limited settings, but also readily available.

This viral infection may be associated with higher mortality and morbidity in pregnant women because of the respiratory, cardiovascular, and immunological changes during pregnancy [2,3]. Pregnant women may be considered to be similar with patients with comorbidities such as asthma, hypertension, and diabetes mellitus [24]. Similar to vulnerable groups with asthma, hypertension, and diabetes mellitus, pregnant patients with COVID-19 disease may experience a more progressive disease because of the oxygen demand and physiological changes in pregnancy. Acute respiratory distress syndrome (ARDS), a type of inflammatory response, is characterized with increased pulmonary edema and endothelial injury [25]. ARDS is the most common reason for COVID-19-related deaths. This viral-infection-related increased inflammatory response was shown in several studies [25,26]. The reduction in the inflammatory response by using steroids may decrease the risk of the occurrence of ARDS and may stop the progression of this lung disease [26,27]. Another worrisome situation with the increased risk in the pregnant women with COVID-19 disease is preterm labor [7]. Preterm delivery may be related to the increased maternal inflammatory response in this disease. Inadequate fetal pulmonary development is a serious health problem especially for preterm newborns. In the current study, the mean hospital stay was 6 days in Group 1, 9 days in Group 2, and 11 days in Group 3. Untreated pregnant women with COVID-19 had the highest rate of ICU admission due to respiratory deterioration. Because pregnant women with COVID-19 have an accentuated risk of hospital admission and intensive care, they are likely to develop ARDS—and, when they do, clinicians must use the corticosteroid that poses the least risk to both the mother and her fetus [8].

In the recent studies and guidelines, the importance of steroid administration in COVID-19 disease has been recognized and different treatment modalities have been presented [7,9,17]. While fetal growth restriction, neonatal glucose regulation, and maternal complications are the main reasons why the use of corticosteroids is rare in pregnant women, steroid therapy is very important for fetal pulmonary development in pregnant women at risk of preterm delivery [10,11]. In the current study, the frequency of increased blood glucose levels related to steroid administration was similar in the groups. Murphy et al. [28] highlight that the placenta contains a distinct enzyme that metabolizes several corticosteroids. The placenta, however, metabolizes more prednisolone and betamethasone than beclomethasone and dexamethasone. It implies that the placenta helps protect the fetus from the corticosteroid side effects through this extensive metabolism to inactive products. Therefore, prednisolone becomes a viable option [28].

The aim of applying a short-course early steroid treatment protocol is to reduce the risk of ARDS occurrence, and to support a better fetal lung development in preterm delivery that may occur due to COVID-19. We observed less oxygen support requirement and a lower occurrence of ARDS in Group 1, and no preterm newborn with respiratory distress in the groups. According to our data, in this study, starting steroid treatment brought positive results in pregnant women, while starting it earlier increased the effectiveness of treatment. Villar et al. [29] reported that early steroid treatment in patients with ARDS reduces the duration of mechanical ventilation. Although steroid treatment has positive effects on lung disease, prolonged treatment may lead to undesirable side effects such as insufficient fetal development [10,11]. In our study, we observed the need for steroid treatment for fewer days in Group 1 compared to the other groups, and early-onset steroid treatment may be more acceptable due to the possibility of a less-side-effects profile with fewer days of drug use. Wang et al. [30] reported in a review that they strongly supported the administration of dexamethasone as a pharmaceutical treatment in pregnant women with COVID-19 before a better treatment was developed. However, they also suggested that the possible negative side effects should not be ignored. 

In the current study, there was no statistically significant difference between the groups in the rate of preterm labor, low birth weight, average birth weight, and decrease in baby movements. There are experimental studies and clinical observations reporting that prenatal exposure to methylprednisolone may be associated with fetal growth restriction and low birth weight [31,32]. However, in some cases, it is also thought that fetal restriction is frequently related to an underlying collagen vascular disease [33]. Methylprednisolone and other glucocorticoids are also indicated in the treatment of neonatal respiratory distress syndrome caused by premature birth, and are successfully used [34,35]. The RECOVERY trial deduced that low-dose dexamethasone (6 mg) reduced mortality by up to one-third among COVID-19 patients on mechanical ventilation and one-fifth among those receiving supplemental oxygen. However, it showed no benefit among COVID-19 patients with mild disease [36]. 

The strengths of this study are the appropriate sample size, reliable data on study objectives, and the low number of deaths. The present study has certain limitations. Firstly, a case series is subject to selection bias because the researcher selects the cases himself. Due to insufficient hospital records, those who gave birth after discharge were not included in the study. The variability in the types of steroids used, variations in the time of administration, and variations in CT imaging are other limitations of the study. 

## 5. Conclusions

There are very limited studies on the impact of COVID-19 infection, especially on pregnant women and newborns. The use of steroids in pregnant women with COVID-19 is a protocol applied in the case of certain criteria, and studies on this subject are limited and unclear. It is important that pregnant patients are admitted to the ICU early after the diagnosis of critical illness and evaluated with a multidisciplinary approach together with an obstetrician. In this study, it was observed that early-onset treatment may be beneficial for fewer hospitalizations, fewer ICU stays, and less mechanical ventilation requirement. Another important point is that no death was observed in this treatment method. In addition, with early treatment, the total number of steroid administration days has been reduced, which is important in terms of reducing the risk of side effects. These results support the continued use of systemic dexamethasone in hospitalized pregnant women with COVID-19. Regarding the management of COVID-19 infection, there is no definitive evidence-based guideline specific to pregnant patients, but multicentre studies are needed.

## Figures and Tables

**Table 1:** Clinical features of the patients at admission to hospital.

	Group 1 n:112 (44.3%)	Group 2 n:90 (35.6%)	Group 3 n:51 (20.2%)	*p*
Clinical stage				0.609
Advanced	47 (92.2%)	106 (94.6%)	82 (91.1%)	
Moderate	4 (7.8%)	6 (5.4%)	8 (8.9%)	
CT grade				0.349
Early	5 (4.5%)	8 (8.9%)	4 (7.8%)	
Progression	99 (88.4%)	70 (77.8%)	43 (84.3%)	
Peak	8 (7.1%)	12 (13.3%)	4 (7.8%)	
Maternal Age	29.1 ± 6.4	31.1 ± 6.0	29.7 ± 6.0	0.139
BMI (kg/m^2^)	33.1 ± 3.8	34.3 ± 3.9	33.5 ± 4.1	0.134
Duration between symptom onset–hospital admission (day)	8.3 ± 3.0	7.8 ± 3.0	8.0 ± 2.5	0.633
Gestational age (week)	31.7 ± 6.8	29.5 ± 7.7	28.6 ± 10.9	0.085

Group 1: Treatment started at admission to hospital; Group 2: Treatment started after 24th hour after admission; Group 3: No steroid treatment; CT: computed tomography; BMI: body mass index.

**Table 2:** Maternal and neonatal outcomes for each treatment in COVID-19-positive pregnant women.

	Group 1 n:112 (44.3%)	Group 2 n:90 (35.6%)	Group 3 n:51 (20.2%)	*p*
Intensive care unit stay (day)	2.7 ± 0.4 ^α,β^	5.7 ± 1.3	8.2 ± 5.5	0.005
Total hospital stay (day)	6.0 ± 1.1 ^α,β^	8.8 ± 1.9 ^γ^	11.1 ± 3.9	<0.001
Newborn weight	3004 ± 625	3108 ± 581	3263 ± 394	0.358
APGAR score (5 min)	8.1 ± 0.7	8.4 ± 0.6	7.9 ± 0.6	0.094
Total steroid days	4.4 ±1.3	5.7 ± 1.4		<0.001
Respiratory support (n)				0.006
HFO	3 (2.7%)	4 (4.4%)	3 (5.9%)	
NIMV	3 (2.7%)	12 (13.3%)	8 (15.7%)	
IMV	1 (0.9%)	3 (3.3%)	4 (7.8%)	
Maternal hyperglycemia (n)	10 (8.9%)	10 (11.1%)	0	0.606
Preterm labor (n)	17 (34%)	9 (33.3%)	4 (33.3%)	0.998
Low birth weight (n)	10 (20%)	6 (22.2%)	2 (16.7%)	0.992
Baby with decreased movements (n)	6 (12%)	3 (11.1%)	1 (8.3%)	

Group 1: Treatment started at admission to hospital; Group 2: Treatment started after 24th hour after admission; Group 3: No steroid treatment; APGAR: appearance, pulse, grimace, activity, respiration; HFO: high-flow oxygen, NIMV; non-invasive mechanical ventilation, IMV: invasive mechanical ventilation. ^α^: Statistically significant difference between Group 1 and 2; ^β:^ Statistically significant difference between Group 1 and 3; ^γ^: Statistically significant difference between Group 2 and 3.

## Data Availability

The data underlying this article are available in the article. If needed, please contact the corresponding author at the following email address: huzun59@hotmail.com.
